# The Potential Role of Retinol-Binding Protein 4 in Heart Failure: A Review

**DOI:** 10.31083/RCM40127

**Published:** 2025-09-26

**Authors:** Jiayi Liu, Yaping Wang

**Affiliations:** ^1^Department of Cardiology, The Second Affiliated Hospital, School of Medicine, Zhejiang University, 310009 Hangzhou, Zhejiang, China; ^2^State Key Laboratory of Transvascular Implantation Devices, 310009 Hangzhou, Zhejiang, China; ^3^Heart Regeneration and Repair Key Laboratory of Zhejiang Province, 310009 Hangzhou, Zhejiang, China

**Keywords:** retinol-binding protein 4, heart failure, inflammation, oxidative stress

## Abstract

Heart failure (HF) is a heterogeneous clinical syndrome, the prevalence of which is increasing among younger adults, promoting global concern due to its significant morbidity and mortality. Therefore, predicting the occurrence of HF using risk-related biomarkers is essential for screening and prevention. Retinol-binding protein 4 (RBP4) is a 21 kDa secreted factor produced by the liver and adipose tissue. Elevated serum RBP4 levels​ are consistently observed in HF patients and are associated with different New York Heart Association (NYHA) class and left ventricular dysfunction. In addition to its role in retinol transport, emerging evidence suggests that RBP4 contributes to the pathogenesis of HF by inducing insulin resistance, triggering chronic inflammation, and directly injuring cardiomyocytes. Studies have found that RBP4 is a potential diagnostic biomarker for HF; however, its clinical relevance is limited due to a paucity of clinical studies and basic science research. This article reviews the current clinical and experimental evidence regarding the pathophysiological effects of RBP4 related to its role in the progression of HF.

## 1. Introduction

Heart failure (HF) impacts approximately 56 million individuals globally and is increasing due to an aging population with increased risk factors. Moreover, young adults (15–44 years) are experiencing an increase in mortality (age-adjusted rate: 3.16 in 2019) compared to older adults (75+ years). Meanwhile, data from Europe and North America indicate a significant rise in HF with preserved ejection fraction (HFpEF), especially among women [1, 2]. Currently, the diagnosis of HF primarily relies on a Clinical Triad as reflected in several well-established diagnostic systems, such as the Framingham criteria, which may not consistently provide appropriate sensitivity and specificity data [3]. The European Society of Cardiology (ESC) guidelines are more accurate and are established on evidence-based medicine (e.g., integration of N-terminal pro-brain natriuretic peptide and imaging criteria). However, these guidelines are limited in providing explicit recommendations for the clinical utilization of emerging biomarkers [4, 5].

Retinol-binding protein 4 (RBP4), a plasma secreted protein, is primarily released by the liver and adipose tissue. RBP4 is recognized mainly for its crucial role in transporting vitamin A, which is essential for maintaining vision, immunity, skin health, and cellular processes [6]. Additionally, the involvement of RBP4 in insulin resistance and systemic inflammation has been extensively documented. Recent research has highlighted that RBP4 exhibits cardiac-specific effects, including myocardial fibrosis and hypertrophy, which are mediated by the Toll-like receptor 4 (TLR4)/myeloid differentiation primary response 88 (MYD88) pathway [7, 8]. Furthermore, RBP4 levels have been shown to increase significantly, by 2–3 fold, in the serum of HF patients compared to healthy controls [9], and are negatively correlated with left ventricular shortening fraction (LVFS) and ejection fraction (LVEF) (*p* < 0.05) [10]. Elevated RBP4 levels are strongly linked to chronic HF progression and adverse outcomes [11]. Therefore, we propose that RBP4 intensifies HF through metabolic–inflammatory interactions, and may represent a new biomarker for risk assessment. However, current studies on RBP4 in HF are limited by inadequate control for confounding factors, insufficient sample sizes, and a lack of understanding of its mechanisms of action. Standardized diagnostic thresholds and consensus on their pathophysiological roles across HF phenotypes (HFrEF/HFpEF) have not been established. Thus, this article aims to review the diagnostic function of RBP4 and delineate its involvement in the etiology of cardiomyocyte dysfunction.

## 2. Retinol-Binding Protein 4

### 2.1 Structure, Metabolism, and Transport Function

**Fig. 1.**
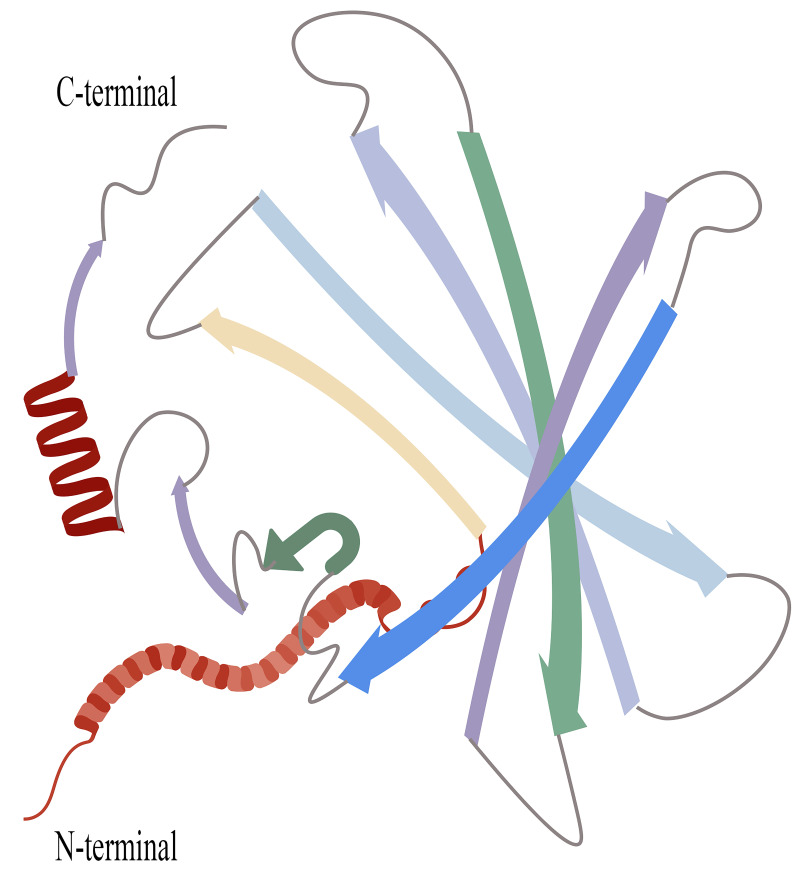
**Structure of RBP4**. Created with BioGDP.com. The eight arrows on the right side of the diagram represent the eight antiparallel β-strands of RBP4, which collectively form a canonical β-barrel structure, creating a hydrophobic core cavity that binds the retinol molecule. The C-terminus (red α-helix) and N-terminus are positioned on either side of the β-barrel, contributing to the maintenance of the overall conformational stability of RBP4. RBP4, retinol-binding protein 4.

RBP4 belongs to the lipocalin family and is a 21 kDa plasma protein first described by Masamitsu Kanai in 1968 [12]. Kanai *et al*. [12] conducted a study examining the plasma of patients who had received radiolabeled retinol by injection and were able to isolate and purify the protein bound to the labeled retinol, which was named RBP4, and is a single polypeptide chain consisting of 201 amino acids and three disulfide bridges in humans [13]. RBP4 performs as a lipocalin-fold tertiary structure, which contains a N-terminal loop, a core β-barrel structure, and a C-terminal alpha-helix [14]. The core structure of RBP4 is a β-barrel. Indeed, RBP4 is composed of eight antiparallel β-strands, featuring a central cavity with four tryptophan residues (W24, W67, W91, and W105) that accommodate the retinol hydrophobic amino acid residues in a trimethylcyclohexenyl ring and an isoprene tail. Additionally, R139 (arginine 139) in the β-barrel is postulated to mediate intermolecular interactions with partner proteins, such as transthyretin (TTR) [15]. The N-terminal coil and the C-terminal alpha-helix are usually located on the outside of the molecule and are responsible for protein stability and interactions, as detailed in Fig. 1 [16].

The circulating RPB4 concentration is ~2–3 µM in humans and ~1 µM in mice [17]. The hepatocytes are the primary site responsible for the synthesis of RBP4, and mature adipocytes are the secondary site, secreting approximately 20% [18]. Mice with hepatic *RBP4* gene-specific knockout have been shown to possess undetectable serum levels of RBP4, proving that adipocyte RBP4 is not a significant source of circulating RBP4 in mice, even in the setting of insulin resistance [19]. In addition, human kidneys, retinal pigment epithelial cells, peritubular cells of the testis, Sertoli cells, spleen, brain, stomach, small intestine, pancreas, and choroid plexus have different levels of *RBP4* mRNA [20, 21].

RBP4 has a critically important role in retinol homeostasis *in vivo*, whereby RBP4 is responsible for transporting retinol from the liver to peripheral tissues; furthermore, circulating levels of RBP4 are positively correlated with the concentration of retinol [22]. There are two main forms of RBP4 involved in retinol transport in the blood. The one bound to retinol is called holo-RBP4, and is the main form of RBP4 found under normal physiological conditions. Holo-RBP4 is highly lipophilic and is responsible for transporting vitamin A to peripheral tissues, especially retinal and epithelial tissues, after its synthesis in the liver. The form not bound to retinol is called apo-RBP4 and normally accounts for only 13–17% of the total RBP4. The ratio of apo-RBP4 in the blood may be increased in the presence of reduced retinol levels (e.g., vitamin A deficiency) or disturbed RBP4 metabolism [16]. When RBP4 function is impaired, the uptake of LPL-mediated lipoprotein-derived retinyl esters may be an important alternative source of cellular retinoids [23].

RBP4 homeostasis relies on binding to the plasma thyroxine transport protein (TTR) in the endoplasmic reticulum (ER) endomorphism [24, 25]. The TTR monomer also features eight antiparallel β-strands, ensuring that the monomer forms a TTR tetramer–retinol transporter complex with RBP4 in a 1:1 ratio, creating a 76 kDa complex [26, 27]. This complex has no significant effect on systemic retinoid homeostasis, even in the presence of TTR deficiency. This may be due to the compensatory transport function of other carrier proteins, such as albumin [28]. The presence of this complex significantly prevents the protein from being filtered out of the proximal tubules of the kidney as the free RBP4 form, thereby maintaining its plasma concentration [29]. Additionally, more than 99% can be reabsorbed via the megalin–cubilin receptor complex. In patients with chronic renal failure, RBP4 is present in two forms. In addition to the 21 kDa full-length protein described above, another protein lacks one or both of the C-terminal amino acids, leucine, which can be removed by healthy patients [30]. RBP4 also has other chemical transportation functions. Crystallographic studies have shown that host-derived oleic and linoleic acids, as well as certain fatty acids, are present in the binding pocket of RBP4 [31].

There are two main RBP4 receptors involved in retinol metabolism: those stimulated by retinoic acid 6 (STRA6) and the others stimulated by retinoic acid 6-like (STRA6L). STRA6 is mainly expressed in extrahepatic tissues, with the highest expression in the retinal pigment epithelium (RPE) [32, 33]. The function of STRA6 in bidirectional transport is mediated by esterification via lecithin: retinol acyltransferase (LRAT) [34]. When binding with RBP4, STRA6 can facilitate the uptake of extrahepatic cells and transport retinol from holo-RBP4, where intracellular retinol binds to cellular retinol-binding protein 1 (CRBP1) for storage and metabolism. The prerequisite is that holo-RBP4 must be isolated from the TTR to allow the assembly with the STRA6 dimer, which subsequently enters the lipophilic cleft [35, 36]. Mutations in the *STRA6* gene can lead to the Matthew-Wood syndrome, the main features of which include microphthalmia, pulmonary hypoplasia [2], heart defects, and diaphragmatic hernias [37]. STRA6 can also impair insulin signaling by enabling tyrosine phosphorylation to activate Janus kinase 2 (JAK2)/signal transducer and the activator of transcription 5 (STAT5) signaling pathway. STRA6L, also known as retinol-binding protein 4 receptor 2 (RBP4R2), represents the main receptor in the liver and intestine, and shares 20% homology with STRA6. Notably, STRA6L is mainly responsible for the retinol uptake by hepatocytes. Meanwhile, studies have shown a possible negative feedback mechanism in regulating STRA6L expression through hepatic retinol storage capacity [38, 39]. SYL residues (S294, Y295, and L296) in mouse RBPR2 have been previously shown to be important for RBP4–retinol (ROL) binding and retinol uptake using in vitro and CRISPR mutant zebrafish models [40]. Both receptors regulate the abundance of retinoids *in vivo*, indirectly affecting the activation of the retinoic acid receptor (RAR)/retinoid X receptor (RXR) complex and influencing their gene transcription and expression, which subsequently modulate the effects of retinoids on cell proliferation, differentiation, and immune responses [28]. However, the effect of retinol status on RBP4 levels has not been uniformly verified, particularly regarding its species-specific effects. Soprano *et al*. [41] confirmed in animal experiments that hepatic RBP4 levels did not differ significantly in rats with altered retinol concentrations. In contrast, a study by Hermsdorff *et al*. [42] in non-obese Spanish women showed a positive correlation between vitamin A intake and RBP4 concentrations. Therefore, further studies are warranted to elucidate the ​interaction mechanisms​ among ​retinol, RBP4, and their cognate receptors.

### 2.2 Regulation of RBP4 Gene Expression and Single Nucleotide Polymorphisms​

The *RBP4* gene is located at 10q23.33, with a full length of 10,050 bp, and contains six exons and five introns in its mRNA, which is approximately 1070 bp in length [43]. The regulation of *RBP4* gene expression is a multilevel process.

At the transcriptional level, hepatocyte nuclear factor 1 alpha (HNF1A) is an important transcription factor in the synthesis of RBP4 in hepatocytes. HNF1A binds to a specific site in the 5^′^ side region of the *RBP4* gene responsible for the high hepatic transcriptional promoter region, and recruits other co-activators and RNA polymerases that activate *RBP4* gene transcription [44]. In a study by Munkhtulga *et al*. [45], rs3758539, i.e., -803 G>A, a functional single-nucleotide polymorphism (SNP) located 5 bp downstream of the HNF1A binding site, affected the binding efficiency of HNF1A and increased RBP4 transcriptional activity. In adipocytes, the peroxisome proliferator-activated receptor gamma (PPARγ) plays an important role in regulating *RBP4* gene expression. PPARγ and RXR can be activated by PPARγ agonists and 9-cis retinoic acid, forming a heterodimer that binds to the PPAR response element (PPRE) in the promoter region of the *RBP4* gene. The heterodimer can either recruit co-activators (e.g., SRC-1, CBP/p300) to promote gene expression through histone acetylation or transcriptional co-repressors (e.g., NCoR, SMRT) to repress transcription through the deacetylation of histones [46]. However, the net effect of PPARγ activation on RBP4 expression remains controversial: Pioglitazone treatment increased adipose tissue RBP4 mRNA in patients with impaired glucose tolerance (IGT), whereas plasma RBP4 levels remained unchanged, suggesting tissue-specific post-transcriptional regulation [47]. cAMP can also activate RBP4 expression through distinct tissue-specific mechanisms involving both transcriptional and translational regulation. In hepatocytes, cAMP–PKA signaling upregulates HMGA1 expression, which facilitates the recruitment of a multiprotein complex containing steroidogenic factor 1 (SF1) to the RBP4 promoter [48], thereby enhancing its transcriptional activity [49]. In brown adipocytes, cAMP has also been shown to regulate *RBP4* gene expression via PPARγcoactivator-1α (PGC-1α) [50]. Additionally, post-transcriptional control is achieved through nutrient-activated mechanistic target of rapamycin complex 1 (mTORC1) signaling, which promotes the translation of RBP4 mRNA in hepatocytes independently of transcriptional regulation [51].

There are several post-translational modifications (PTMs) of RBP4. Indeed, methylation of RBP4 was first reported in an esophageal cancer model, where treatment with the demethylating agent 5-aza-2^′^-deoxycytidine (aza-dC) restored RBP4 expression, confirming its role in epigenetic silencing [52]. Phosphorylation of RBP4 also plays an important role in several biological processes. Notably, phosphorylated RBP4 has been shown to promote denervation-induced muscle atrophy and correlates with the elevated expression of muscle atrophy markers such as atrogin-1 and MuRF1, via the STRA6/JAK2/STAT3 pathway [53]. These coordinated mechanisms ensure precise spatiotemporal control of RBP4 production.

Hormonal and adipokine regulation also significantly influences RBP4 levels. A well-established bidirectional relationship exists between insulin resistance and elevated RBP4 levels [54]. Furthermore, adipose-derived hormones such as leptin and lipocalin modulate RBP4 levels. Multivariate regression analyses have identified leptin as a positive predictor of RBP4 in both subcutaneous and visceral adipose tissue in women; meanwhile, lipocalin serves as a predictive factor for RBP4 specifically in male visceral adipose tissue [55]. Conversely, experimental evidence demonstrates that atrial natriuretic peptide (ANP) directly modulates the secretory activity of adipose tissue, thereby reducing the generation of RBP4 [56]. Nonetheless, the specific molecular pathways through which these neuroendocrine factors regulate *RBP4* gene expression await further investigation.

There are numerous SNPs associated with a high risk of cardiovascular disease (CVD) in different regions of the *RBP4* gene. In a study among Spanish children, rs3758538, located in the 5^′^ flanking region of the first promoter, and rs12265684, located in the non-coding region between exons 4 and 5, were shown to be associated with blood pressure. Mean arterial pressure was higher in minor allele *C* carriers than in major allele *G* carriers [57]. In another study, rs7094671, another non-coding region located between exons 4 and 5, was confirmed to be associated with an increased risk of developing CAD. The *A* allele was more suggestive than the *G* allele, but this SNP was not confirmed to be related to the severity of CAD in this study [58, 59]. Some SNPs may be associated with the protection of patients with CVD, but the mechanisms underlying this association remain incompletely understood. In a study of a Chinese Han population, the minor *C* allele rs3758538 was significantly associated with a lower risk of hypertriglyceridemia. The Shanghai subgroup with minor *G* allele rs17108993 showed a lower risk of hypertensive disease [60].

## 3. Role of Retinol-Binding Protein 4 in Heart Failure

### 3.1 Potential Biomarker Role of Retinol-Binding Protein 4 in Heart Failure: An Overview of Available Clinical Studies

Several studies have demonstrated that RBP4 is a promising biomarker for predicting HF and related adverse events. In a prospective cohort study in older adults with chronic heart failure (CHF), serum RBP4 concentration showed a positive correlation with New York Heart Association (NYHA) classification guidelines and a negative correlation with LVEF (*p* < 0.01). LogRBP4 was considered to be an independent predictor of cardiovascular mortality (hazard ratio (HR) = 2.24, 95% confidence interval (CI) = 1.35–5.39; *p* < 0.01) and CHF rehospitalization (HR = 2.54, 95% CI = 1.09–5.60; *p* < 0.01) [61], and negatively correlated with renal function (r = –0.159; *p* < 0.001) [9].

#### 3.1.1 Retinol-Binding Protein 4 Levels Associated With Risk and Protective Factors of Heart Failure

Recent research indicates that the circulating levels of RBP4 are substantially influenced by modifiable lifestyle factors, which may, in turn, affect the risk of HF. Elevated RBP4 concentrations have been consistently shown to correlate with several metabolic and behavioral risk factors. Specifically, obesity and proinflammatory dietary patterns, particularly high-fat/high-carbohydrate Western diets, have been demonstrated to elevate RBP4​​ concentrations significantly in clinical models [62]. Additionally, research conducted by Gao *et al*. [63] involving normoglycemic healthy males, as well as a cross-sectional study by Hong *et al*. [64], indicated that smoking tobacco and chronic alcohol consumption are independent behavioral risk factors linked to elevated RBP4 levels. This diet-induced dysregulation of RBP4 exacerbates insulin resistance and systemic inflammatory responses, ultimately accelerating the pathophysiology and progression of HF [65].

Sleep and mental disorders have also been linked to raised RBP4 levels, which are positively correlated with a heightened incidence of HF. Obstructive sleep apnea syndrome (OSAS) is a chronic inflammatory condition that results in upper airway obstruction during sleep. Untreated OSAS causes HF and other CVDs [66]. RBP4 was found to exhibit a positive correlation with the apnea–hypopnea index (AHI) in a study of OSAS patients (r = 0.47; *p* < 0.01). Shorter sleep duration and irregular sleep periods are also associated with higher RBP4 levels, increasing the risk of HF [67, 68]. Previous studies have also shown that depression contributes to the risk of HF [69]. RBP4 was also observed to be associated with age, the onset and duration of major depressive disorders [70].

In addition to the aforementioned risk factors, several studies have demonstrated that ​RBP4 levels​ are also ​significantly associated with​ various ​cardioprotective behaviors [71], as detailed in Table 1 (Ref. [63, 64, 67, 68, 70, 72, 73, 74, 75, 76, 77, 78, 79]). Resistance training​ and ​high total physical activity levels​ have been shown to reduce ​circulating RBP4 concentrations [72]. Similarly, specific ​dietary patterns, such as the ​Mediterranean and ​ketogenic diets [73], demonstrate comparable effects. These findings not only support the role of RBP4 as a ​potential biomarker​ for ​HF risk but also suggest its utility as a ​modifiable preventive target, highlighting its ​dual function​ in ​lifestyle intervention strategies.

**Table 1.** **Retinol-binding protein 4 in patients with risk and protective factors**.

Factors or condition		Year (Ref)	Study design	Study population	Correlation	Study size	Analysis
Risk factors	Cigarette smoking	2012 [63]	Cross-sectional analysis	Healthy male subjects with normal glucose tolerance	+	136	Correlation
	Chronic alcohol intake	2022 [64]	Cross-Sectional Study	Han Chinese adults aged >18 years	+	2075	Correlation
	Sedentary lifestyle	2018 [74]	Cross-sectional study	Sedentary T2D patients	+	106	Multivariate regression
	High salt intake	2021 [75]	Cross-sectional study	Healthy Chinese subjects	+	42	Correlation
	Low AHI	2023 [76]	Case–control study	OSAS group and HC	+	171	Correlation
	Irregular sleep habits	2023 [67]	Cross-sectional study	Workers from the OHSPIW cohort	+	1499	Multivariate regression
	Shorter sleep duration	2017 [68]	Cross-sectional study​	School-aged children	+	3166	Multivariate regression
	Mental disorder	2020 [70]	Case–control study	MDD patients and HC	+	285	Correlation
	Obesity	2022 [77]	Cohort study	Non-diabetic participants aged ≥40 years	+	784	Correlation
Protective factors	Resistance exercise	2010 [72]	Cohort study	Female patients with T2D	–	44	Correlation
	High levels of total physical activity	2009 [78]	Cross-sectional study	Chinese people aged 50–70 years	–	3289	Multivariate regression
	Ketogenic diet	2023 [73]	Cohort study	Middle-aged male patients with metabolic syndrome	–	40	Correlation
	Low-fat diet	2016 [79]	Cross-sectional study	Patients with hypertriglyceridemia	–	46	Correlation

+, positive correlation; –, negative correlation; T2D, type 2 diabetes; OSAS, obstructive sleep apnea syndrome; AHI, apnea–hypopnea index; MDD, major depressive disorder; HC, health control.

#### 3.1.2 RBP4 Related Biological Risk Factors and Heart Diseases

Beyond the prognostic value of RBP4, these studies have shown its significant associations with biological risk factors and heart diseases related to HF, as detailed in Table 2 (Ref. [9, 10, 80, 81, 82, 83, 84, 85, 86, 87, 88, 89, 90]).

**Table 2.** **Retinol-binding protein 4 in patients with biological risk factors and heart diseases**.

Factors or condition	Year (Ref)	Study design	Study population	Positive correlation event	Study size	Analysis
CHF	2020 [80]	Cohort study	CHF patients aged ≥60 years and HC	Cardiovascular mortality re-hospitalization	1072	Multivariate regression
	2018 [9]	Cross-sectional analysis	participants of the PolSenior study aged ≥65 years	Renal function	2826	Correlation
Hypertension	2022 [81]	Case–cohort study	EPIC-Potsdam cohort	Cardiometabolic risk	27,548	Multivariate regression
	2024 [82]	Cross-sectional study	EH with T2D and HC	Disease state	119	Correlation
	2019 [10]	Cross-sectional study	EH and HC	E/A on echocardiogram	120	Correlation
Hyperlipidemia	2018 [83]	Cross-sectional study	Participants in the PolSenior study aged ≥65 years	Hypertriglyceridemia, hypertension	3038	Multivariate regression
	2018 [84]	Cohort study	School-aged children	Triglyceride concentration, insulin resistance	352	Multivariate regression
Diabetes mellitus	2022 [85]	Case–control study	DCM and HC	Disease state	347	Multivariate analysis
Coronary artery disease	2020 [86]	Case–control study	ACS patients and non-CAD patients	CAD diagnosis	240	Multivariate regression
	2023 [87]	Cross-sectional study	T2D patients with/without CHD	Coronary artery wall elastic	130	Multivariate regression
	2022 [88]	Cohort study	Patients with stable CAD	MACEs	840	Multivariate regression
	2021 [89]	Case–control study	ACS patients and patients with cardiovascular risk factors but normal angiography	CAD severity	98	Correlation
Cardiomyopathy	2017 [90]	Cohort study	Patients with ATTR	ATTR diagnosis	111	Multivariate regression

CHF, chronic heart failure; HC, health control; EPIC-Potsdam cohort, European Prospective Investigation into Cancer and Nutrition-Potsdam cohort; EH, essential hypertension; T2D, type 2 diabetes; DCM, diabetic cardiomyopathy; ACS, acute coronary syndrome; CHD, coronary heart disease; CAD, coronary artery disease; MACEs, major adverse cardiovascular events; ATTR, amyloid transthyretin; OHSPIW cohort, Occupational Health Study of Petroleum Industry Workers cohort.

3.1.2.1 Components of Metabolic Syndrome

Metabolic syndrome (MetS) is a collection of interconnected metabolic disorders (including obesity, hypertension, dyslipidemia, and insulin resistance) that collectively raise the risk of CVD and type 2 diabetes mellitus (T2DM) [91]. RBP4 was observed to be associated with multiple components of MetS, and elevated RBP4 levels in childhood are also good predictors of their cardiometabolic risk in adults [84].

RBP4 exhibits different characteristics in hypertensive patients of different genders, disease stages, and therapy. Plasma RBP4 concentration was significantly higher in the male hypertension population than in normotensive patients (median concentration [95% CI]: 43.4 [30.4–64.8] vs. 38.1 [27.1–54.4] ng/mL, respectively; *p* < 0.01); however, this difference was only significant in female patients taking four or more antihypertensive drugs [92]. A study showed that RBP4 was also elevated in patients with pre-hypertension (pre-HT) and positively correlated with body mass index (BMI), systolic blood pressure (SBP), and diastolic blood pressure (DBP) (r = 0.226, 0.468, 0.358, respectively; all *p* ≤ 0.001) [93].

In patients with diabetic cardiomyopathy, both RBP4 levels showed a positive linear association with the risk of diabetic DCM (odds ratio (OR) = 16.87 (6.5, 43.23); *p* < 0.001), even after adjusting for confounding variables [85].

3.1.2.2 Vascular Disease

Among patients with CAD, the RBP4 concentration showed a positive correlation with small, dense low-density lipoprotein (sd-LDL) levels (r = 0.273; *p* = 0.001) and oxidized low-density lipoprotein (ox-LDL) levels (r = 0.167; *p* = 0.043). This suggests that RBP4 may play an important role in atherosclerosis, particularly in the formation of sd-LDL [59]. Additionally, RBP4 has been linked to vascular function and clinical prognosis. In patients with coronary heart disease (CHD) and T2DM, RBP4 is an independent risk factor for the coronary artery elasticity parameter β (coefficient 1.330 (0.909–1.751); *p* = 0.031), even after adjusting for the effects of age and pulse pressure [87, 94]. In patients with acute coronary syndrome (ACS), RBP4, in combination with a scoring system consisting of NT-proBNP, LVEF, estimated glomerular filtration rate (eGFR), and age, predicted the risk of major adverse cardiovascular events (MACEs) (*p* < 0.05 for each component), and its increased levels have been correlated with the severity of CAD [89, 95].

3.1.2.3 Cardiomyopathy

Amyloid transthyretin (ATTR) cardiomyopathy is a cause of HF in older adults that has been attributed to mutant TTR proteins or RBP4. Indeed, RBP4 was shown to be a predictor of ATTR in conjunction with LVEF, interventricular septal wall thickness, and mean limb lead voltage in a cohort study, with a threshold value of <49.5 µg/mL, and was a highly sensitive predictor of V122I ATTR (AUC = 0.92 (0.86–0.99) [90].

### 3.2 Mechanism of RBP4 in Heart Failure

#### 3.2.1 Effects of RBP4 on Cardiac Metabolism

RBP4 has been found to play a crucial role in the energy metabolism abnormalities associated with HF by regulating fatty acid metabolism, glucose utilization, and insulin signaling pathways through autocrine or paracrine signaling [96].

3.2.1.1 RBP4 Mediates Systemic Insulin Resistance Leading to Heart Failure

Optimal cardiac function requires a consistent supply of adenosine triphosphate (ATP) from two primary sources: mitochondrial oxidative phosphorylation and glycolysis, which require coordination between cardiomyocytes and the circulatory system [97]. Under normal conditions, the main source of energy for the myocardium is fatty acids (≈40% to 60%), with glucose and other substrates, such as lactate, serving as alternative substrates [98]. In the failing heart, insulin resistance (IR) affects both insulin-mediated glucose uptake and the direct activation of glucose oxidation by insulin, leading to metabolic disorders and adverse effects on left ventricular remodeling [99]. RBP4 contributes to HF by mediating multiple metabolic disorders, including insulin resistance [7].

RBP4-mediated insulin resistance can be categorized into two pathways: retinol-dependent and retinol-independent. The retinol–RBP4 complex mediates insulin resistance mainly through interaction with the STRA6 receptor, subsequently activating the JAK2/STAT5 suppressor of cytokine signaling 3 (SOCS3) signaling pathway. SOCS3 specifically inhibits the binding of Phosphoinositide 3-kinase (PI3K) to insulin receptor substrate (IRS)-1 by increasing its serine phosphorylation in the PI3K/Protein kinase B (AKT) pathway, leading to IR, which is mainly found in adipose tissues [61]. In the retinoid-independent mechanism, RBP4 increases the hepatic expression of phosphoenolpyruvate carboxykinase (PEPCK), which catalyzes the conversion of oxaloacetate to phosphoenolpyruvate, thereby increasing glucose production by gluconeogenesis in the liver. The chronic hyperglycemic state stimulates pancreatic β-cells to compensate by secreting excessive amounts of insulin, ultimately leading to the development of insulin resistance and compensatory hyperinsulinemia [61]. RBP4 activates antigen-presenting cells via JNK–TLR4 signaling, triggering the release of proinflammatory cytokines (TNF-α and interleukin (IL)-6) that establish a chronic low-grade inflammatory state, which impairs insulin signaling [100, 101]. These tissue-specific mechanisms synergistically amplify RBP4-induced systemic insulin resistance.

Glucose transporter 4 (GLUT4) expression is decreased in adipocytes in nearly all insulin-resistant states in humans and rodents. In a study by Yang *et al*. [7], adipose-specific deletion of glucose transporter-4 (adipose-*GLUT4*-/-) mice exhibited increased levels of *RBP4* mRNA and serum protein, which increased PI3K activity by 80% in muscle tissue and interrupted insulin signaling. Insulin signaling in the heart is crucial for regulating myocardial metabolism of oxidative substrates, specifically glucose and fatty acids. Insulin resistance leads to a reduction in glucose oxidation in myocardial cells, either by decreasing glucose uptake or by directly inhibiting mitochondrial pyruvate dehydrogenase (PDH) activity [102]. This results in decreased myocardial glucose metabolism (MrGlu) and ATP production from glucose metabolism, subsequently leading to myocardial contractile dysfunction, resulting in decreased myocardial mechanical energy efficiency (MEEi), ultimately leading to myocardial hypertrophy and diastolic dysfunction. This is the primary mechanism through which RBP4 mediates HFpEF via insulin resistance [103]. During HFrEF, there is uncoupling between glycolysis and glucose oxidation, causing acidosis, which worsens contractile dysfunction in the failing heart by desensitizing contractile proteins to Ca^2+^, slowing the inward Ca^2+^ current, and redirecting cardiac ATP to ionic homeostasis instead of contractility [104]. Myocardial compensation mechanisms are dependent on excess fatty acid oxidation to maintain ATP production [105]. However, a study demonstrated that liver-specific RBP4 overexpression did not impair glucose homeostasis and whole-body energy metabolism in mice. This finding differs from other prior studies, which showed impairment of glucose homeostasis in mice with muscle-specific and adipose-specific overexpression of RBP4 [106, 107]. Thus, it was hypothesized that endogenous RBP4 protein levels may exhibit a distinct expression or secretion pattern, as well as a varying degree of retinol binding, compared to the overexpressed protein. The RBP4 secreted by the liver does not affect local RBP4 functions in adipose tissue and, therefore, fails to affect glucose homeostasis. Thus, the mechanism through which RBP4 mediates HF through insulin resistance requires further clarification [108].

In addition, RBP4 also enhances insulin-induced proliferation of RASMCs and the expression of p-ERK1/2 and p-JAK2, which can be inhibited by ERK1/2 and vitamin D inhibitors but not by JAK2 inhibitors [109, 110]. This mechanism may also result in HF.

3.2.1.2 RBP4-Mediated Heart Failure via Lipid Metabolism Disorders

In HF, while fatty acid oxidation (FAO) remains the primary source of ATP, a discrepancy can occur between lipid uptake and utilization in cardiomyocytes [111]. The production of malonyl-CoA is elevated, functioning as an allosteric inhibitor of carnitine palmitoyl transferase in various cell types, including cardiomyocytes, thereby restricting the transfer of fatty acids into mitochondria [112]. PPARs serve as the principal regulators of cardiac fatty acid metabolism, with PPARα exhibiting the highest expression in cardiomyocytes [113]. RBP4 diminishes PPARα activity, decreasing the expression of genes involved in fatty acid oxidation [114]. However, recent data have demonstrated that cardiac FAO can increase in HFpEF; however, the link with RBP4 remains unclear [115].

Intracellular lipids accumulate in HF patients primarily in the form of triglycerides (TGs), diacylglycerols (DAGs), ceramides, cholesterol, and its derivatives. Among these, ceramides and DAGs function as lipotoxic mediators, playing a role in cardiac lipotoxicity [116]. These two lipids change the structure of cell membranes, which directly leads to the death of cardiomyocytes and HFrEF [117]. RBP4 interferes with the anti-lipolytic function of insulin, increasing basal lipolysis and leading to the excessive release of free fatty acids (FFAs), which can be converted into DAG [118]. These DAGs are also associated with the acute induction of insulin resistance by temporally activating protein kinase (PKC)θ, phosphorylating the IRS-1 serine 1101 ion, and dephosphorylating insulin-stimulated IRS-1 tyrosine and AKT2 [119], which exacerbates HF.

Hypercholesterolemia has also been shown to be associated with a worse prognosis in HF by promoting hypertrophy and fibrosis [120], as demonstrated in hypertensive mouse models where lipoprotein lipase inhibitor P-407 worsened diastolic dysfunction [121]. Additionally, hypercholesterolemia disrupts the liver–heart crosstalk, increasing systemic metabolic dysfunction. Elevated RBP4 levels are associated with increased production of apolipoprotein B (a component of very low-density lipoprotein (VLDL)) [122, 123]. An independent association was observed between RBP4 and the percentage of small HDL particles, as well as between RBP4 and LDL-C, HDL-C, and TGs, suggesting that RBP4 may contribute to the formation of small HDL particles and altered lipoprotein profiles [124]. Elevated RBP4 results in increased cholesterol uptake in macrophages, primarily by influencing scavenger receptors such as CD36 and SR-A1. These receptors facilitate the internalization of ox-LDL, thereby enhancing lipid uptake in macrophages and promoting the formation of foam cells. This process contributes to inflammation in atherosclerotic lesions, which contributes to HF [125].

#### 3.2.2 Effects of RBP4 in Myocardial Injury and Inflammation

Several studies have investigated the impact of inflammatory factors on RBP4 concentrations. Bobbert *et al*. [126] demonstrated that IL-8 enhances adipocyte RBP4 expression in a dose-dependent manner. A positive correlation has also been found between adipose RBP4 and the inflammatory marker CD68 [100]. Though basic research on myocardial injury directly caused by RBP4 remains limited, it has been shown that RBP4 can trigger a decline in cardiac function and ultimately lead to HF by promoting myocardial inflammation, pyroptosis, and cardiomyocyte hypertrophy.

3.2.2.1 RBP4 Induces Myocardial Pyroptosis via the NLRP3/Caspase-1/GSDMD Axis

**Fig. 2.**
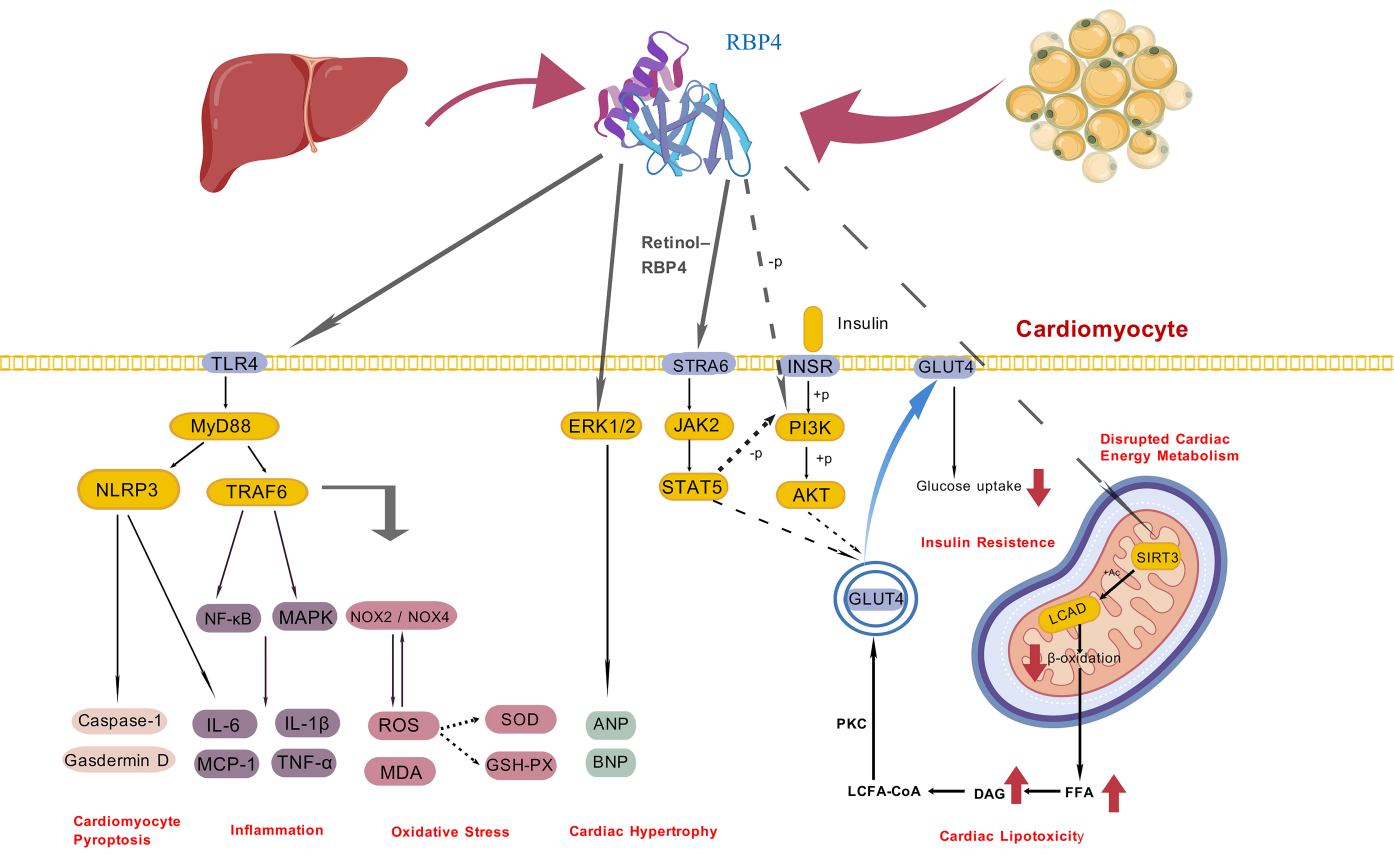
**Mechanism of RBP4 in heart failure**. Created with BioGDP.com. This schematic diagram illustrates the molecular mechanisms through which ​retinol-binding protein 4 (RBP4)​​ contributes to ​heart failure pathogenesis​ through: (1) activation of cardiomyocyte pyroptosis via the TLR/NLRP3 pathway, (2) activation of the cardiomyocyte inflammation via the TLR4/MYD88 pathway, (3) enhanced oxidative stress and generation of ROS, (4) promotion of cardiomyocyte hypertrophy via the MAPK/ERK pathway, (5) exacerbation of insulin resistance by the JAK2/STAT5 and PI3K/AKT pathway, (6) promotion of energy and metabolic lipid disorders. RBP4, retinol-binding protein 4; TLR4, Toll-like receptor 4; STRA6, stimulated by retinoic acid 6; INSR, insulin receptor; GLUT4, glucose transporter type 4; MYD88, myeloid differentiation primary response 88; NLRP3, NOD-like receptor family pyrin domain-containing 3; TRAF6, TNF receptor-associated factor 6; ANP, atrial natriuretic peptide; BNP, brain natriuretic peptide; ERK, extracellular regulated protein kinases; IL, interleukin; MCP-1, monocyte chemoattractant protein-1; NOX, NADPH oxidase; ROS, reactive oxygen species; MDA, malondialdehyde; SOD, superoxide dismutase; DAG, diacylglycerol; LCFA-CoA, long-chain fatty acyl-coenzyme A; PKC, protein kinase C; LCAD, long-chain Acyl-CoA dehydrogenase; FFAs, free fatty acids; SIRT3, sirtuin 3.

In a study by Zhang *et al*. [127], using a mouse model of AMI induced by left anterior descending coronary artery ligation, it was found that in the border zone of infarcted myocardium as well as in ischemia/hypoxia (I/H) -treated mouse primary heart cardiomyocytes, there was a marked increase in the expression of RBP4, and RBP4 activated caspase-1 cleavage through a direct interaction with NOD-like receptor family pyrin domain-containing 3 (NLRP3), which, in turn, induced a gasdermin D (GSDMD) dependent pyroptosis pathway. This process led to cardiomyocyte death and further deterioration of cardiac function. Knockdown of the *RBP4* gene using an adenovirus was found to significantly attenuate the ischemia–hypoxia-induced cardiomyocyte injury and pyroptosis, suggesting a critical role of RBP4 in cardiomyocyte death [127]. CHF after myocardial pyroptosis is commonly the result of prolonged neurohormonal activation and sustained remodeling, in which necrotic tissue is replaced by scar tissue after myocardial infarction resulting in ventricular remodeling, with thinning of the infarcted myocardial wall and enlargement of the left ventricular cavity leading to loss of systolic function and increased wall stress [128], This remodeling process is also exacerbated by the activation of signaling pathways by elevated levels of catecholamines and angiotensin II (Ang II), as shown in Fig. 2 [129, 130].

3.2.2.2 RBP4 Promotes Myocardial Inflammation and Hypertrophy via the TLR4/MYD88 Pathway

In a study by Gao *et al*. [131], a model of cardiac hypertrophy induced by transverse aortic constriction (TAC) and Ang II infusion was constructed in mice. RBP4 levels were found to be significantly higher in the serum TAC group than in the control group. *RBP4* mRNA was selectively increased in white adipose tissue (WAT). In the Ang II group, serum RBP4 levels increased and were positively correlated with Ang II. *In vitro* experiments with RBP4-stimulated cardiomyocytes also showed a dose-dependent increase in cell volume and the level of RBP4, in addition to enhanced expression of inflammatory factors (e.g., TNF-α, IL-6, MCP-1, and IL-1β), TLR4, and MYD88 in cardiomyocytes, which was significantly attenuated by the TLR4 inhibitor, TAK242, and by knockdown of the *MYD88* gene, suggesting that RBP4 induces inflammation and oxidative stress in cardiomyocytes through the activation of the TLR4/MYD88 pathway leading to myocardial hypertrophy and, ultimately, HF [131]. RBP4 mediates the myocardial inflammatory response through TLR4, and this activation also initiates the formation of NLRP3 inflammatory vesicles [132], a key participant in aseptic inflammation [133]. RBP4 triggers the maturation of proinflammatory cytokines (IL-1β and IL-18) to initiate the inflammatory response and plays a key role in altering the physiological state of cardiomyocytes and leading to the progression of HF [134]. In HF with HFpEF, systemic inflammation increases ventricular stiffness by triggering the expression of vascular cell adhesion molecules, recruiting macrophages converted from monocytes, secreting transforming growth factor β, and stimulating collagen deposition by myofibroblasts. Inflammation also leads to a reduction in Titin phosphorylation and an increase in disulfide bond formation, resulting in the hardening of Titin, a giant sarcomere protein, and ultimately, diastolic left ventricular stiffness, which can lead to HF.

#### 3.2.3 Role of RBP4 in Oxidative Stress and Free Radical Generation

Increased oxidative stress is a key factor in the pathogenesis of HF, leading to inflammation, activation of the renin-angiotensin–aldosterone system and the sympathetic nervous system, as well as vascular remodeling and damage to cellular components, which contribute to myocardial dysfunction and the progression of HF [135]. In a study by Wang *et al*. [136], RBP4 levels showed a positive correlation with the oxidative stress marker 8-iso-PGF2α and a negative correlation with antioxidant enzymes such as catalase (CAT), which explains the association between RBP4 and oxidative stress. RBP4 has also been shown to exacerbate oxidative stress in RBP4-Tg mice with reduced mitochondrial DNA content, abnormal mitochondrial ultrastructure, reduced mitochondrial β-oxidase activity, and increased levels of long-chain acyl-coenzyme A dehydrogenase (LCAD) acetylation due to RBP4 overexpression, and inhibition of SIRT3 activity [137]. RBP4 also dose-dependently and significantly upregulated the gene expression of *VCAM-1*, *ICAM-1*, *E-selectin*, and *MCP-1*, increased nuclear translocation and phosphorylation of the NF-κB subunit p65, and enhanced the expression of NADPH oxidase-dependent Nox2 and Nox4 independently of STRA6 [138]. RBP4-induced oxidative stress also interferes with insulin signaling and exacerbates the metabolic syndrome [61]. The modulation of oxidative stress by RBP4 may originate from the induction of mitochondrial dysfunction. Human aortic endothelial cells (HAECs) subjected to different doses of RBP4 showed that an increase in the concentration of RBP4 significantly inhibited the phosphorylation of PI3K at the Ser473 locus, resulting in the downregulation of the expression of *MNF1* (which mediates mitochondrial fusion) genes mediated via the PI3K–AKT pathway. In contrast, the expression of the Drp1 and Fis1 proteins (mediating mitochondrial fission) increased, resulting in a dose-dependent accumulation of mitochondrial superoxide. In contrast, decreased Bcl-2 protein expression and increased cytochrome C and Bax protein expression mediated the deterioration of cardiac function in the arteries of RBP4-Tg mice in the *in vivo* assay [139].

## 4. RBP4 Inhibitors in the Treatment of Heart Failure

In view of the role of RBP4 in facilitating cardiac metabolism, oxidative stress, and myocardial injury, known critical contributors to HF, targeting RBP4 has become a promising therapeutic approach. The following paragraphs summarize the effectiveness of current pharmacological RBP4 inhibitors, metabolic medications, and direct RBP4 antagonists in alleviating the progression of HF.

### 4.1 Metabolic Drugs With Indirect RBP4-Lowering Effects

RBP4 plays a significant role in cardiac metabolism in HF. Consequently, metabolic drugs that alter RBP4 levels may provide valuable therapeutic options for the management of HF.

Oral antidiabetic drugs, such as pioglitazone, a PPARγ agonist commonly used to treat T2DM, have been recently discovered to significantly decrease serum RBP4 levels and body weight in obese rats, leading to an improvement in insulin sensitivity. This could have important implications for the treatment of HF [140]. Another hypoglycemic medication, sitagliptin, a sodium–glucose cotransporter 2 (SGLT2) inhibitor, significantly reduced RBP4 levels in T2DM patients. However, this drug may also increase the metabolic syndrome and the risk of cardiovascular complications in non-diabetic patients [141]. In contrast, pioglitazone (PIO) and simvastatin (SIMVA) do not affect RBP4 levels, although these compounds significantly improve homeostatic model assessment of insulin resistance (HOMA-IR) scores [142].

In a study of CAD patients, rosuvastatin significantly reduced serum RBP4 levels and mitigated disease progression [143]. Wu and colleagues found that in studies on rats and 3T3-L1 adipocytes, fenofibrate decreased adipocyte *RBP4* mRNA levels and improved insulin sensitivity. Treatment with fenofibrate for 8 weeks resulted in a 30% reduction in serum RBP4 concentrations in insulin-resistant and dyslipidemic males [144].

A natural compound, resveratrol, has been shown to protect the heart by decreasing oxidative stress and apoptosis in HF by activating Foxo3a [145]. Meanwhile, resveratrol has also been found to lower RBP4 expression and enhance cardiovascular function in rats [146].

### 4.2 RBP4 Targeting Drugs

Fenretinide, a synthetic retinol derivative, can enter the central cavity of the β-barrel structure of RBP4, preventing its interaction with TTR through spatial site-blocking. A reversible dose-dependent decrease in serum RBP4 was found after fenretinide treatment in a clinical study [147]. There are several novel synthetic antagonists of limited clinical use. A1120, a non-retinoid ligand, reduces plasma RBP4 levels by binding with high affinity to RBP4. However, its poor hepatic microsomal stability limits its current clinical application. BPN-14136, a bicyclic analogue, exhibits high RBP4 binding affinity and microsomal stability, which has been demonstrated in animal studies [13]. A new specific RBP4 antagonist has been developed called aptamer-conjugated calcium phosphate nanoparticles. However, there is currently no evidence of its *in vivo* application in clinical studies [148].

Metabolic drugs may help to decrease RBP4 levels, whereas direct antagonists might protect the heart via other mechanisms. In the future, RBP4-targeted treatments may play an important therapeutic role in HF patients with RBP4-associated pathology.

## 5. Limitations and Future Directions

Although RBP4 is now considered to be associated with HF, current research remains limited. The following points need to be clearly addressed in future studies: First, RBP4 lacks a clear threshold as a biomarker for HF. Large-scale clinical trials are required in the future to establish a standard threshold for RBP4. Second, multiple factors, including nutrition, hormones, and drugs, can affect circulating RBP4 levels. Most existing studies only consider a small portion of these factors, and future clinical research must strictly and comprehensively control for these confounding factors to minimize experimental error and bias. Third, while prior research has elucidated several potential mechanisms through which RBP4 may contribute to HF, the mechanism through which it affects the myocardium remains unclear. Hence, future studies are needed to determine the mechanism for these actions.

## 6. Conclusion

RBP4, a retinol transporter protein in the lipid transport protein family, plays an important role in the diagnosis, prognostic assessment, and pathogenesis of HF. Clinical studies have shown that high RBP4 levels correlate with risk factors and adverse cardiovascular events of HF, revealing its potential value for diagnosis and disease prognosis. The role of RBP4 in the pathogenesis of HF has been studied in metabolic disorders, inflammation, direct myocardial injury, and oxidative stress, which together exacerbate cardiac function. Metabolic drugs and direct RBP4 antagonists also provide some guidance for the treatment of HF. Future research will further elucidate the intricate mechanisms through which RBP4 functions, potentially leading to favorable alterations in the diagnosis and treatment of HF.

## References

[1] Khan MS, Shahid I, Bennis A, Rakisheva A, Metra M, Butler J (2024). Global epidemiology of heart failure. *Nature Reviews. Cardiology*.

[2] Feng J, Zhang Y, Zhang J (2024). Epidemiology and Burden of Heart Failure in Asia. *JACC. Asia*.

[3] McKee PA, Castelli WP, McNamara PM, Kannel WB (1971). The natural history of congestive heart failure: the Framingham study. *The New England Journal of Medicine*.

[4] McDonagh TA, Metra M, Adamo M, Gardner RS, Baumbach A, Böhm M (2024). 2023 Focused update of the 2021 ESC Guidelines for the diagnosis and treatment of acute and chronic heart failure. *Giornale Italiano Di Cardiologia (2006)*.

[5] Gaggin HK, Januzzi JL (2013). Biomarkers and diagnostics in heart failure. *Biochimica et Biophysica Acta*.

[6] Mark M, Ghyselinck NB, Chambon P (2006). Function of retinoid nuclear receptors: lessons from genetic and pharmacological dissections of the retinoic acid signaling pathway during mouse embryogenesis. *Annual Review of Pharmacology and Toxicology*.

[7] Yang Q, Graham TE, Mody N, Preitner F, Peroni OD, Zabolotny JM (2005). Serum retinol binding protein 4 contributes to insulin resistance in obesity and type 2 diabetes. *Nature*.

[8] Olsen T, Blomhoff R (2020). Retinol, Retinoic Acid, and Retinol-Binding Protein 4 are Differentially Associated with Cardiovascular Disease, Type 2 Diabetes, and Obesity: An Overview of Human Studies. *Advances in Nutrition (Bethesda, Md.)*.

[9] Majerczyk M, Choręza P, Mizia-Stec K, Bożentowicz-Wikarek M, Brzozowska A, Arabzada H (2018). Plasma Level of Retinol-Binding Protein 4, N-Terminal proBNP and Renal Function in Older Patients Hospitalized for Heart Failure. *Cardiorenal Medicine*.

[10] Li X, Zhu S, Song G, Zhang K, Gao W, Huang J (2019). Retinol-binding protein 4 is closely correlated to blood pressure level and E/A in untreated essential hypertension patients. *Annals of Palliative Medicine*.

[11] Rafaqat S (2023). Adipokines and Their Role in Heart Failure: A Literature Review. *The Journal of Innovations in Cardiac Rhythm Management*.

[12] Kanai M, Raz A, Goodman DS (1968). Retinol-binding protein: the transport protein for vitamin A in human plasma. *The Journal of Clinical Investigation*.

[13] Kim N, Priefer R (2021). Retinol binding protein 4 antagonists and protein synthesis inhibitors: Potential for therapeutic development. *European Journal of Medicinal Chemistry*.

[14] Steinhoff JS, Lass A, Schupp M (2022). Retinoid Homeostasis and Beyond: How Retinol Binding Protein 4 Contributes to Health and Disease. *Nutrients*.

[15] Greene LH, Chrysina ED, Irons LI, Papageorgiou AC, Acharya KR, Brew K (2001). Role of conserved residues in structure and stability: tryptophans of human serum retinol-binding protein, a model for the lipocalin superfamily. *Protein Science: a Publication of the Protein Society*.

[16] Zanotti G, Berni R (2004). Plasma retinol-binding protein: structure and interactions with retinol, retinoids, and transthyretin. *Vitamins and Hormones*.

[17] Shirakami Y, Lee SA, Clugston RD, Blaner WS (2012). Hepatic metabolism of retinoids and disease associations. *Biochimica et Biophysica Acta*.

[18] Tsutsumi C, Okuno M, Tannous L, Piantedosi R, Allan M, Goodman DS (1992). Retinoids and retinoid-binding protein expression in rat adipocytes. *The Journal of Biological Chemistry*.

[19] Thompson SJ, Sargsyan A, Lee SA, Yuen JJ, Cai J, Smalling R (2017). Hepatocytes Are the Principal Source of Circulating RBP4 in Mice. *Diabetes*.

[20] Naylor HM, Newcomer ME (1999). The structure of human retinol-binding protein (RBP) with its carrier protein transthyretin reveals an interaction with the carboxy terminus of RBP. *Biochemistry*.

[21] Amengual J, Zhang N, Kemerer M, Maeda T, Palczewski K, Von Lintig J (2014). STRA6 is critical for cellular vitamin A uptake and homeostasis. *Human Molecular Genetics*.

[22] Erikstrup C, Mortensen OH, Nielsen AR, Fischer CP, Plomgaard P, Petersen AM (2009). RBP-to-retinol ratio, but not total RBP, is elevated in patients with type 2 diabetes. *Diabetes, Obesity & Metabolism*.

[23] Quadro L, Hamberger L, Gottesman ME, Colantuoni V, Ramakrishnan R, Blaner WS (2004). Transplacental delivery of retinoid: the role of retinol-binding protein and lipoprotein retinyl ester. *American Journal of Physiology. Endocrinology and Metabolism*.

[24] Steinhoff JS, Lass A, Schupp M (2021). Biological Functions of RBP4 and Its Relevance for Human Diseases. *Frontiers in Physiology*.

[25] Bellovino D, Morimoto T, Tosetti F, Gaetani S (1996). Retinol binding protein and transthyretin are secreted as a complex formed in the endoplasmic reticulum in HepG2 human hepatocarcinoma cells. *Experimental Cell Research*.

[26] Campos-Sandoval JA, Redondo C, Kinsella GK, Pal A, Jones G, Eyre GS (2011). Fenretinide derivatives act as disrupters of interactions of serum retinol binding protein (sRBP) with transthyretin and the sRBP receptor. *Journal of Medicinal Chemistry*.

[27] Goodman AB (2006). Retinoid receptors, transporters, and metabolizers as therapeutic targets in late onset Alzheimer disease. *Journal of Cellular Physiology*.

[28] Muenzner M, Tuvia N, Deutschmann C, Witte N, Tolkachov A, Valai A (2013). Retinol-binding protein 4 and its membrane receptor STRA6 control adipogenesis by regulating cellular retinoid homeostasis and retinoic acid receptor α activity. *Molecular and Cellular Biology*.

[29] Prapunpoj P (2009). Evolutionary changes to transthyretin. *The FEBS Journal*.

[30] Raila J, Willnow TE, Schweigert FJ (2005). Megalin-mediated reuptake of retinol in the kidneys of mice is essential for vitamin A homeostasis. *The Journal of Nutrition*.

[31] Perduca M, Nicolis S, Mannucci B, Galliano M, Monaco HL (2018). High resolution crystal structure data of human plasma retinol-binding protein (RBP4) bound to retinol and fatty acids. *Data in Brief*.

[32] Kawaguchi R, Yu J, Honda J, Hu J, Whitelegge J, Ping P (2007). A membrane receptor for retinol binding protein mediates cellular uptake of vitamin A. *Science (New York, N.Y.)*.

[33] Wolf G (2007). Identification of a membrane receptor for retinol-binding protein functioning in the cellular uptake of retinol. *Nutrition Reviews*.

[34] Amengual J, Golczak M, Palczewski K, von Lintig J (2012). Lecithin:retinol acyltransferase is critical for cellular uptake of vitamin A from serum retinol-binding protein. *The Journal of Biological Chemistry*.

[35] Chen Y, Clarke OB, Kim J, Stowe S, Kim YK, Assur Z (2016). Structure of the STRA6 receptor for retinol uptake. *Science (New York, N.Y.)*.

[36] Isken A, Golczak M, Oberhauser V, Hunzelmann S, Driever W, Imanishi Y (2008). RBP4 disrupts vitamin A uptake homeostasis in a STRA6-deficient animal model for Matthew-Wood syndrome. *Cell Metabolism*.

[37] Pasutto F, Sticht H, Hammersen G, Gillessen-Kaesbach G, Fitzpatrick DR, Nürnberg G (2007). Mutations in STRA6 cause a broad spectrum of malformations including anophthalmia, congenital heart defects, diaphragmatic hernia, alveolar capillary dysplasia, lung hypoplasia, and mental retardation. *American Journal of Human Genetics*.

[38] Fedders R, Muenzner M, Schupp M (2015). Retinol binding protein 4 and its membrane receptors: a metabolic perspective. *Hormone Molecular Biology and Clinical Investigation*.

[39] Majerczyk M, Olszanecka-Glinianowicz M, Puzianowska-Kuźnicka M, Chudek J (2016). Retinol-binding protein 4 (RBP4) as the causative factor and marker of vascular injury related to insulin resistance. *Postepy Higieny i Medycyny Doswiadczalnej (Online)*.

[40] Radhakrishnan R, Leung M, Solanki AK, Lobo GP (2023). Mapping of the extracellular RBP4 ligand binding domain on the RBPR2 receptor for Vitamin A transport. *Frontiers in Cell and Developmental Biology*.

[41] Soprano DR, Smith JE, Goodman DS (1982). Effect of retinol status on retinol-binding protein biosynthesis rate and translatable messenger RNA level in rat liver. *The Journal of Biological Chemistry*.

[42] Hermsdorff HHM, Zulet MÁ, Abete I, Martínez JA (2009). Discriminated benefits of a Mediterranean dietary pattern within a hypocaloric diet program on plasma RBP4 concentrations and other inflammatory markers in obese subjects. *Endocrine*.

[43] Kotnik P, Fischer-Posovszky P, Wabitsch M (2011). RBP4: a controversial adipokine. *European Journal of Endocrinology*.

[44] Colantuoni V, Pirozzi A, Blance C, Cortese R (1987). Negative control of liver-specific gene expression: cloned human retinol-binding protein gene is repressed in HeLa cells. *The EMBO Journal*.

[45] Munkhtulga L, Nakayama K, Utsumi N, Yanagisawa Y, Gotoh T, Omi T (2007). Identification of a regulatory SNP in the retinol binding protein 4 gene associated with type 2 diabetes in Mongolia. *Human Genetics*.

[46] Tontonoz P, Spiegelman BM (2008). Fat and beyond: the diverse biology of PPARgamma. *Annual Review of Biochemistry*.

[47] Yao-Borengasser A, Varma V, Bodles AM, Rasouli N, Phanavanh B, Lee MJ (2007). Retinol binding protein 4 expression in humans: relationship to insulin resistance, inflammation, and response to pioglitazone. *The Journal of Clinical Endocrinology and Metabolism*.

[48] Chiefari E, Paonessa F, Iiritano S, Le Pera I, Palmieri D, Brunetti G (2009). The cAMP-HMGA1-RBP4 system: a novel biochemical pathway for modulating glucose homeostasis. *BMC Biology*.

[49] Jessen KA, Satre MA (1998). Induction of mouse retinol binding protein gene expression by cyclic AMP in Hepa 1-6 cells. *Archives of Biochemistry and Biophysics*.

[50] Rosell M, Hondares E, Iwamoto S, Gonzalez FJ, Wabitsch M, Staels B (2012). Peroxisome proliferator-activated receptors-α and -γ, and cAMP-mediated pathways, control retinol-binding protein-4 gene expression in brown adipose tissue. *Endocrinology*.

[51] Neyestani TR, Nikooyeh B, Alavi-Majd H, Shariatzadeh N, Kalayi A, Tayebinejad N (2012). Improvement of vitamin D status via daily intake of fortified yogurt drink either with or without extra calcium ameliorates systemic inflammatory biomarkers, including adipokines, in the subjects with type 2 diabetes. *The Journal of Clinical Endocrinology and Metabolism*.

[52] Tsunoda S, Smith E, De Young NJ, Wang X, Tian ZQ, Liu JF (2009). Methylation of CLDN6, FBN2, RBP1, RBP4, TFPI2, and TMEFF2 in esophageal squamous cell carcinoma. *Oncology Reports*.

[53] Zhang KZ, Li JW, Xu JS, Shen ZK, Lin YS, Zhao C (2024). RBP4 promotes denervation-induced muscle atrophy through STRA6-dependent pathway. *Journal of Cachexia, Sarcopenia and Muscle*.

[54] Graham TE, Yang Q, Blüher M, Hammarstedt A, Ciaraldi TP, Henry RR (2006). Retinol-binding protein 4 and insulin resistance in lean, obese, and diabetic subjects. *The New England Journal of Medicine*.

[55] Bianconcini A, Lupo A, Capone S, Quadro L, Monti M, Zurlo D (2009). Transcriptional activity of the murine retinol-binding protein gene is regulated by a multiprotein complex containing HMGA1, p54 nrb/NonO, protein-associated splicing factor (PSF) and steroidogenic factor 1 (SF1)/liver receptor homologue 1 (LRH-1). *The International Journal of Biochemistry & Cell Biology*.

[56] Moro C, Klimcakova E, Lolmède K, Berlan M, Lafontan M, Stich V (2007). Atrial natriuretic peptide inhibits the production of adipokines and cytokines linked to inflammation and insulin resistance in human subcutaneous adipose tissue. *Diabetologia*.

[57] Codoñer-Franch P, Carrasco-Luna J, Allepuz P, Codoñer-Alejos A, Guillem V (2016). Association of RBP4 genetic variants with childhood obesity and cardiovascular risk factors. *Pediatric Diabetes*.

[58] Wan K, Zhao J, Deng Y, Chen X, Zhang Q, Zeng Z (2014). A genetic polymorphism in RBP4 is associated with coronary artery disease. *International Journal of Molecular Sciences*.

[59] Wu J, Shi YH, Niu DM, Li HQ, Zhang CN, Wang JJ (2012). Association among retinol-binding protein 4, small dense LDL cholesterol and oxidized LDL levels in dyslipidemia subjects. *Clinical Biochemistry*.

[60] Wu Y, Li H, Loos RJF, Qi Q, Hu FB, Liu Y (2009). RBP4 variants are significantly associated with plasma RBP4 levels and hypertriglyceridemia risk in Chinese Hans. *Journal of Lipid Research*.

[61] Flores-Cortez YA, Barragán-Bonilla MI, Mendoza-Bello JM, González-Calixto C, Flores-Alfaro E, Espinoza-Rojo M (2022). Interplay of retinol binding protein 4 with obesity and associated chronic alterations (Review). *Molecular Medicine Reports*.

[62] Zabetian-Targhi F, Mahmoudi MJ, Rezaei N, Mahmoudi M (2015). Retinol binding protein 4 in relation to diet, inflammation, immunity, and cardiovascular diseases. *Advances in Nutrition (Bethesda, Md.)*.

[63] Gao S, Wang YH, Li M (2012). Cigarette smoking increases levels of retinol-binding protein-4 in healthy men with normal glucose tolerance. *Chinese Medical Journal*.

[64] Hong GB, Shao XF, Li JM, Zhou Q, Ke XS, Gao PC (2022). Associaton of Retinol Binding Protein 4 (RBP4) Levels With Hyperuricemia: A Cross-Sectional Study in a Chinese Population. *Frontiers in Endocrinology*.

[65] Fan J, Hu J (2024). Retinol binding protein 4 and type 2 diabetes: from insulin resistance to pancreatic β-cell function. *Endocrine*.

[66] Polecka A, Olszewska N, Pulido M, Olszewska E (2024). Rostral fluid shifts and other mechanisms of interaction between obstructive sleep apnea and heart failure - a systematic review. *Otolaryngologia Polska = the Polish Otolaryngology*.

[67] Wang L, Ma Q, Fang B, Su Y, Lu W, Liu M (2023). Shift work is associated with an increased risk of type 2 diabetes and elevated RBP4 level: cross sectional analysis from the OHSPIW cohort study. *BMC Public Health*.

[68] Li L, Fu J, Yu XT, Li G, Xu L, Yin J (2017). Sleep Duration and Cardiometabolic Risk Among Chinese School-aged Children: Do Adipokines Play a Mediating Role?. *Sleep*.

[69] Burg MM (2021). Depression and Heart Failure: What Then Must We Do?. *JACC. Heart Failure*.

[70] Yao Q, Li Y (2020). Study of decreased serum levels of retinol binding protein 4 in major depressive disorder. *Journal of Psychiatric Research*.

[71] Phillips A, Cobbold C (2014). A Comparison of the Effects of Aerobic and Intense Exercise on the Type 2 Diabetes Mellitus Risk Marker Adipokines, Adiponectin and Retinol Binding Protein-4. *International Journal of Chronic Diseases*.

[72] Ku YH, Han KA, Ahn H, Kwon H, Koo BK, Kim HC (2010). Resistance exercise did not alter intramuscular adipose tissue but reduced retinol-binding protein-4 concentration in individuals with type 2 diabetes mellitus. *The Journal of International Medical Research*.

[73] Ghorbanian B, Wong A, Iranpour A (2023). The effect of dietary carbohydrate restriction and aerobic exercise on retinol binding protein 4 (RBP4) and fatty acid binding protein 5 (FABP5) in middle-aged men with metabolic syndrome. *The British Journal of Nutrition*.

[74] Klisic A, Kavaric N, Ninic A (2018). Retinol-binding protein 4 versus albuminuria as predictors of estimated glomerular filtration rate decline in patients with type 2 diabetes. *Journal of Research in Medical Sciences: the Official Journal of Isfahan University of Medical Sciences*.

[75] Liu F, Zhang R, Zhang W, Zhu L, Yu Q, Liu Z (2021). Potassium supplementation blunts the effects of high salt intake on serum retinol-binding protein 4 levels in healthy individuals. *Journal of Diabetes Investigation*.

[76] Fazlıoğlu N, Uysal P, Durmus S, Yurt S, Gelisgen R, Uzun H (2023). Significance of Plasma Irisin, Adiponectin, and Retinol Binding Protein-4 Levels as Biomarkers for Obstructive Sleep Apnea Syndrome Severity. *Biomolecules*.

[77] Xiang J, Dai H, Hou Y, Wang Q, Wang T, Li M (2022). Sexual Dimorphism in the Association of Serum Retinol-Binding Protein-4 With Long-Term Dynamic Metabolic Profiles in Non-Diabetes. *Frontiers in Endocrinology*.

[78] Yu Z, Ye X, Wang J, Qi Q, Franco OH, Rennie KL (2009). Associations of physical activity with inflammatory factors, adipocytokines, and metabolic syndrome in middle-aged and older chinese people. *Circulation*.

[79] Christou GA, Tellis CC, Elisaf MS, Tselepis AD, Kiortsis DN (2016). The relationship between retinol-binding protein 4 and apolipoprotein B-containing lipoproteins is attenuated in patients with very high serum triglycerides: A pilot study. *Hormones (Athens, Greece)*.

[80] Li XZ, Zhang KZ, Yan JJ, Wang L, Wang Y, Shen XY (2020). Serum retinol-binding protein 4 as a predictor of cardiovascular events in elderly patients with chronic heart failure. *ESC Heart Failure*.

[81] Schiborn C, Weber D, Grune T, Biemann R, Jäger S, Neu N (2022). Retinol and Retinol Binding Protein 4 Levels and Cardiometabolic Disease Risk. *Circulation Research*.

[82] Jadhao AG, Gaikwad KB, Yadav RR (2024). Serum retinol binding protein 4 in individuals with essential hypertension and type 2 diabetes: A cross-sectional study. *Journal of Family Medicine and Primary Care*.

[83] Majerczyk M, Kocełak P, Choręza P, Arabzada H, Owczarek AJ, Bożentowicz-Wikarek M (2018). Components of metabolic syndrome in relation to plasma levels of retinol binding protein 4 (RBP4) in a cohort of people aged 65 years and older. *Journal of Endocrinological Investigation*.

[84] Li G, Esangbedo IC, Xu L, Fu J, Li L, Feng D (2018). Childhood retinol-binding protein 4 (RBP4) levels predicting the 10-year risk of insulin resistance and metabolic syndrome: the BCAMS study. *Cardiovascular Diabetology*.

[85] Shan H, Ji Y, Gu H, Li H, Zhu J, Feng Y (2022). Elevated Serum Retinol Binding Protein 4 is Associated with the Risk of Diabetic Cardiomyopathy. *Reviews in Cardiovascular Medicine*.

[86] Si Y, Liu J, Han C, Wang R, Liu T, Sun L (2020). The correlation of retinol-binding protein-4 and lipoprotein combine index with the prevalence and diagnosis of acute coronary syndrome. *Heart and Vessels*.

[87] Ji Y, Du S, Tang C, Song J, Gu X (2023). The Value of RBP4 in Assessing Coronary Artery Elasticity in Patients with Coronary Heart Disease and Type 2 Diabetes Mellitus. *Reviews in Cardiovascular Medicine*.

[88] Qian K, Yan X, Xu C, Fang Y, Ma M (2022). Association Between Circulating Retinol-Binding Protein 4 and Adverse Cardiovascular Events in Stable Coronary Artery Disease. *Frontiers in Cardiovascular Medicine*.

[89] Nar G, Sanlialp SC, Nar R (2021). Retinol binding protein 4 levels relate to the presence and severity of coronary artery disease. *Journal of Medical Biochemistry*.

[90] Arvanitis M, Koch CM, Chan GG, Torres-Arancivia C, LaValley MP, Jacobson DR (2017). Identification of Transthyretin Cardiac Amyloidosis Using Serum Retinol-Binding Protein 4 and a Clinical Prediction Model. *JAMA Cardiology*.

[91] Pigeot I, Ahrens W (2025). Epidemiology of metabolic syndrome. *Pflugers Archiv: European Journal of Physiology*.

[92] Majerczyk M, Choręza P, Bożentowicz-Wikarek M, Brzozowska A, Arabzada H, Owczarek A (2017). Increased plasma RBP4 concentration in older hypertensives is related to the decreased kidney function and the number of antihypertensive drugs-results from the PolSenior substudy. *Journal of the American Society of Hypertension: JASH*.

[93] Zhang JX, Zhu GP, Zhang BL, Cheng YY (2017). Elevated serum retinol-binding protein 4 levels are correlated with blood pressure in prehypertensive Chinese. *Journal of Human Hypertension*.

[94] Jugnam-Ang W, Pannengpetch S, Isarankura-Na-Ayudhya P, Thippakorn C, Isarankura-Na-Ayudhya C, Lawung R (2015). Retinol-binding protein 4 and its potential roles in hypercholesterolemia revealed by proteomics. *EXCLI Journal*.

[95] Ye B, Zhao Q, Fan J, Li X, Shan C, Liu F (2023). RBP4-based Multimarker Score: A Prognostic Tool for Adverse Cardiovascular Events in Acute Coronary Syndrome Patients. *The Journal of Clinical Endocrinology and Metabolism*.

[96] Lee SA, Yuen JJ, Jiang H, Kahn BB, Blaner WS (2016). Adipocyte-specific overexpression of retinol-binding protein 4 causes hepatic steatosis in mice. *Hepatology (Baltimore, Md.)*.

[97] Lopaschuk GD, Karwi QG, Tian R, Wende AR, Abel ED (2021). Cardiac Energy Metabolism in Heart Failure. *Circulation Research*.

[98] Gandoy-Fieiras N, Gonzalez-Juanatey JR, Eiras S (2020). Myocardium Metabolism in Physiological and Pathophysiological States: Implications of Epicardial Adipose Tissue and Potential Therapeutic Targets. *International Journal of Molecular Sciences*.

[99] He H, Huang W, Pan Z, Wang L, Yang Z, Chen Z (2025). Intercellular Mitochondrial transfer: Therapeutic implications for energy metabolism in heart failure. *Pharmacological Research*.

[100] Moraes-Vieira PM, Yore MM, Dwyer PM, Syed I, Aryal P, Kahn BB (2014). RBP4 activates antigen-presenting cells, leading to adipose tissue inflammation and systemic insulin resistance. *Cell Metabolism*.

[101] Norseen J, Hosooka T, Hammarstedt A, Yore MM, Kant S, Aryal P (2012). Retinol-binding protein 4 inhibits insulin signaling in adipocytes by inducing proinflammatory cytokines in macrophages through a c-Jun N-terminal kinase- and toll-like receptor 4-dependent and retinol-independent mechanism. *Molecular and Cellular Biology*.

[102] Olefsky JM (1976). Insulin’s effect on glucose oxidation independent of glucose transport. *Biochemical and Biophysical Research Communications*.

[103] Succurro E, Cicone F, Papa A, Miceli S, Vizza P, Fiorentino TV (2023). Impaired insulin-stimulated myocardial glucose metabolic rate is associated with reduced estimated myocardial energetic efficiency in subjects with different degrees of glucose tolerance. *Cardiovascular Diabetology*.

[104] Karwi QG, Uddin GM, Ho KL, Lopaschuk GD (2018). Loss of Metabolic Flexibility in the Failing Heart. *Frontiers in Cardiovascular Medicine*.

[105] Sun Q, Güven B, Wagg CS, Almeida de Oliveira A, Silver H, Zhang L (2024). Mitochondrial fatty acid oxidation is the major source of cardiac adenosine triphosphate production in heart failure with preserved ejection fraction. *Cardiovascular Research*.

[106] Berry DC, Jin H, Majumdar A, Noy N (2011). Signaling by vitamin A and retinol-binding protein regulates gene expression to inhibit insulin responses. *Proceedings of the National Academy of Sciences of the United States of America*.

[107] Cioffi CL, Racz B, Varadi A, Freeman EE, Conlon MP, Chen P (2019). Design, Synthesis, and Preclinical Efficacy of Novel Nonretinoid Antagonists of Retinol-Binding Protein 4 in the Mouse Model of Hepatic Steatosis. *Journal of Medicinal Chemistry*.

[108] Fedders R, Muenzner M, Weber P, Sommerfeld M, Knauer M, Kedziora S (2018). Liver-secreted RBP4 does not impair glucose homeostasis in mice. *The Journal of Biological Chemistry*.

[109] Li F, Xia K, Sheikh MSA, Cheng J, Li C, Yang T (2014). Retinol binding protein 4 promotes hyperinsulinism induced proliferation of rat aortic smooth muscle cells. *Molecular Medicine Reports*.

[110] Zhou W, Wang W, Yuan XJ, Xiao CC, Xing Y, Ye SD (2022). The Effects of RBP4 and Vitamin D on the Proliferation and Migration of Vascular Smooth Muscle Cells via the JAK2/STAT3 Signaling Pathway. *Oxidative Medicine and Cellular Longevity*.

[111] Da Dalt L, Cabodevilla AG, Goldberg IJ, Norata GD (2023). Cardiac lipid metabolism, mitochondrial function, and heart failure. *Cardiovascular Research*.

[112] Wang W, Zhang L, Battiprolu PK, Fukushima A, Nguyen K, Milner K (2019). Malonyl CoA Decarboxylase Inhibition Improves Cardiac Function Post-Myocardial Infarction. *JACC. Basic to Translational Science*.

[113] Prosdocimo DA, John JE, Zhang L, Efraim ES, Zhang R, Liao X (2015). KLF15 and PPARα Cooperate to Regulate Cardiomyocyte Lipid Gene Expression and Oxidation. *PPAR Research*.

[114] Finck BN, Kelly DP (2006). PGC-1 coactivators: inducible regulators of energy metabolism in health and disease. *The Journal of Clinical Investigation*.

[115] De Jong KA, Lopaschuk GD (2017). Complex Energy Metabolic Changes in Heart Failure With Preserved Ejection Fraction and Heart Failure With Reduced Ejection Fraction. *The Canadian Journal of Cardiology*.

[116] Choi RH, Tatum SM, Symons JD, Summers SA, Holland WL (2021). Ceramides and other sphingolipids as drivers of cardiovascular disease. *Nature Reviews. Cardiology*.

[117] Stratford S, Hoehn KL, Liu F, Summers SA (2004). Regulation of insulin action by ceramide: dual mechanisms linking ceramide accumulation to the inhibition of Akt/protein kinase B. *The Journal of Biological Chemistry*.

[118] Lair B, Laurens C, Van Den Bosch B, Moro C (2020). Novel Insights and Mechanisms of Lipotoxicity-Driven Insulin Resistance. *International Journal of Molecular Sciences*.

[119] Szendroedi J, Yoshimura T, Phielix E, Koliaki C, Marcucci M, Zhang D (2014). Role of diacylglycerol activation of PKCθ in lipid-induced muscle insulin resistance in humans. *Proceedings of the National Academy of Sciences of the United States of America*.

[120] Bastien M, Poirier P, Lemieux I, Després JP (2014). Overview of epidemiology and contribution of obesity to cardiovascular disease. *Progress in Cardiovascular Diseases*.

[121] Williams M, Kamiar A, Condor Capcha JM, Rasmussen MA, Alitter Q, Kanashiro Takeuchi R (2024). A Murine Model of Hyperlipidemia-Induced Heart Failure with Preserved Ejection Fraction. *Journal of Visualized Experiments: JoVE*.

[122] Vergès B, Guiu B, Cercueil JP, Duvillard L, Robin I, Buffier P (2012). Retinol-binding protein 4 is an independent factor associated with triglycerides and a determinant of very low-density lipoprotein-apolipoprotein B100 catabolism in type 2 diabetes mellitus. *Arteriosclerosis, Thrombosis, and Vascular Biology*.

[123] Liu Y, Chen H, Wang J, Zhou W, Sun R, Xia M (2015). Elevated retinol binding protein 4 induces apolipoprotein B production and associates with hypertriglyceridemia. *The Journal of Clinical Endocrinology and Metabolism*.

[124] Rocha M, Bañuls C, Bellod L, Rovira-Llopis S, Morillas C, Solá E (2013). Association of serum retinol binding protein 4 with atherogenic dyslipidemia in morbid obese patients. *PloS One*.

[125] Liu Y, Zhong Y, Chen H, Wang D, Wang M, Ou JS (2017). Retinol-Binding Protein-Dependent Cholesterol Uptake Regulates Macrophage Foam Cell Formation and Promotes Atherosclerosis. *Circulation*.

[126] Bobbert P, Weithäuser A, Andres J, Bobbert T, Kühl U, Schultheiss HP (2009). Increased plasma retinol binding protein 4 levels in patients with inflammatory cardiomyopathy. *European Journal of Heart Failure*.

[127] Zhang KZ, Shen XY, Wang M, Wang L, Sun HX, Li XZ (2021). Retinol-Binding Protein 4 Promotes Cardiac Injury After Myocardial Infarction Via Inducing Cardiomyocyte Pyroptosis Through an Interaction With NLRP3. *Journal of the American Heart Association*.

[128] Jenča D, Melenovský V, Stehlik J, Staněk V, Kettner J, Kautzner J (2021). Heart failure after myocardial infarction: incidence and predictors. *ESC Heart Failure*.

[129] Oka T, Akazawa H, Naito AT, Komuro I (2014). Angiogenesis and cardiac hypertrophy: maintenance of cardiac function and causative roles in heart failure. *Circulation Research*.

[130] Jiang S, Li H, Zhang L, Mu W, Zhang Y, Chen T (2025). Generic Diagramming Platform (GDP): a comprehensive database of high-quality biomedical graphics. *Nucleic Acids Research*.

[131] Gao W, Wang H, Zhang L, Cao Y, Bao JZ, Liu ZX (2016). Retinol-Binding Protein 4 Induces Cardiomyocyte Hypertrophy by Activating TLR4/MyD88 Pathway. *Endocrinology*.

[132] Moraes-Vieira PM, Yore MM, Sontheimer-Phelps A, Castoldi A, Norseen J, Aryal P (2020). Retinol binding protein 4 primes the NLRP3 inflammasome by signaling through Toll-like receptors 2 and 4. *Proceedings of the National Academy of Sciences of the United States of America*.

[133] Abbate A (2013). The heart on fire: inflammasome and cardiomyopathy. *Experimental Physiology*.

[134] Paraskevaidis I, Farmakis D, Papingiotis G, Tsougos E (2023). Inflammation and Heart Failure: Searching for the Enemy-Reaching the Entelechy. *Journal of Cardiovascular Development and Disease*.

[135] Chawla HV, Singh N, Singh SB (2024). The Association Between Oxidative Stress and the Progression of Heart Failure: A Systematic Review. *Cureus*.

[136] Wang Q, Tian S, Xiao D, Zhao R, Zhang X, Dou Z (2022). Correlation of serum RBP4 level with oxidative stress and unstable carotid plaque in patients with cerebral infarction. *Translational Neuroscience*.

[137] Liu Y, Mu D, Chen H, Li D, Song J, Zhong Y (2016). Retinol-Binding Protein 4 Induces Hepatic Mitochondrial Dysfunction and Promotes Hepatic Steatosis. *The Journal of Clinical Endocrinology and Metabolism*.

[138] Farjo KM, Farjo RA, Halsey S, Moiseyev G, Ma JX (2012). Retinol-binding protein 4 induces inflammation in human endothelial cells by an NADPH oxidase- and nuclear factor kappa B-dependent and retinol-independent mechanism. *Molecular and Cellular Biology*.

[139] Wang J, Chen H, Liu Y, Zhou W, Sun R, Xia M (2015). Retinol binding protein 4 induces mitochondrial dysfunction and vascular oxidative damage. *Atherosclerosis*.

[140] Zhu C, Xiao Y, Liu X, Han J, Zhang J, Wei L (2015). Pioglitazone lowers serum retinol binding protein 4 by suppressing its expression in adipose tissue of obese rats. *Cellular Physiology and Biochemistry: International Journal of Experimental Cellular Physiology, Biochemistry, and Pharmacology*.

[141] Sun X, Zhang Z, Ning H, Sun H, Ji X (2017). Sitagliptin down-regulates retinol-binding protein 4 and reduces insulin resistance in gestational diabetes mellitus: a randomized and double-blind trial. *Metabolic Brain Disease*.

[142] Pfützner A, Schöndorf T, Hanefeld M, Lübben G, Kann PH, Karagiannis E (2009). Changes in insulin resistance and cardiovascular risk induced by PPARgamma activation have no impact on RBP4 plasma concentrations in nondiabetic patients. *Hormone and Metabolic Research*.

[143] Wang Y, Zhou C, Yu T, Zhao F (2021). Correlation between Changes in Serum RBP4, hs-CRP, and IL-27 Levels and Rosuvastatin in the Treatment of Coronary Heart Disease. *Journal of Healthcare Engineering*.

[144] Wu H, Wei L, Bao Y, Lu J, Huang P, Liu Y (2009). Fenofibrate reduces serum retinol-binding protein-4 by suppressing its expression in adipose tissue. *American Journal of Physiology. Endocrinology and Metabolism*.

[145] Liu K, Zhu Y, Gao W, Han X, Zhang Q, Zhao Y (2024). Resveratrol alleviates heart failure by activating foxo3a to counteract oxidative stress and apoptosis. *Biomedicine & Pharmacotherapy*.

[146] Delucchi F, Berni R, Frati C, Cavalli S, Graiani G, Sala R (2012). Resveratrol treatment reduces cardiac progenitor cell dysfunction and prevents morpho-functional ventricular remodeling in type-1 diabetic rats. *PloS One*.

[147] Dobri N, Qin Q, Kong J, Yamamoto K, Liu Z, Moiseyev G (2013). A1120, a nonretinoid RBP4 antagonist, inhibits formation of cytotoxic bisretinoids in the animal model of enhanced retinal lipofuscinogenesis. *Investigative Ophthalmology & Visual Science*.

[148] Torabi R, Ghourchian H, Amanlou M, Pasalar P (2017). Aptamer-Conjugated Calcium Phosphate Nanoparticles for Reducing Diabetes Risk via Retinol Binding Protein 4 Inhibition. *Canadian Journal of Diabetes*.

